# Investigation of Antiparasitic Activity of Two Marine Natural Products, Estradiol Benzoate, and Octyl Gallate, on *Toxoplasma gondii In Vitro*

**DOI:** 10.3389/fphar.2022.841941

**Published:** 2022-03-17

**Authors:** Daiqiang Lu, Nian-Zhang Zhang, Yinning Yao, Tingting Wang, Qianqian Hua, Xiaozi Zheng, Wei Cong, Feng Tan

**Affiliations:** ^1^ School of Basic Medical Sciences, Wenzhou Medical University, Wenzhou, China; ^2^ State Key Laboratory of Veterinary Etiological Biology, National Animal Echinococcosis Para-Reference Laboratory, Key Laboratory of Veterinary Parasitology of Gansu Province, Lanzhou Veterinary Research Institute, CAAS, Lanzhou, China; ^3^ Clinical Laboratory, Dongyang People’s Hospital, Jinhua, China; ^4^ Marine College, Shandong University, Weihai, China

**Keywords:** *Toxoplasma gondii*, estradiol benzoate, octyl gallate, cell cycle, marine natural product

## Abstract

Toxoplasmosis, caused by *Toxoplasma gondii*, is a common disease worldwide and could be severe and even fatal in immunocompromised individuals and fetuses. Limitation in current available treatment options drives the need to develop novel therapeutics. This study assessed the anti-*T. gondii* potential of 103 marine natural products. A luminescence-based β-galactosidase activity assay was used to screen the marine natural products library. Afterward, those compounds that displayed over 70% parasite inhibition ratio were further chosen to assess their cytotoxicity. Compounds exhibiting low cytotoxicity (≥80% cell viability) were applied to evaluate the inhibition efficacy on discrete steps of the *T. gondii* lytic cycle, including invasion, intracellular growth, and egress abilities as well as the cell cycle. We found that both estradiol benzoate and octyl gallate caused >70% inhibition of tachyzoite growth with IC_50_ values of 4.41 ± 0.94 and 5.66 ± 0.35 μM, respectively, and displayed low cytotoxicity with TD_50_ values of 34.11 ± 2.86 and 26.4 ± 0.98 μM, respectively. Despite their defects in inhibition of invasion and egress of tachyzoite, the two compounds markedly inhibited the tachyzoite intracellular replication. Flow cytometric analyses further suggested that the anti-*T. gondii* activity of estradiol benzoate, rather than octyl gallate, may be linked to halting cell cycle progression of tachyzoite from G1 to S phase. Taken together, these findings suggest that both estradiol benzoate and octyl gallate are potential inhibitors for anti-*T. gondii* infection and support the further exploration of marine natural products as a thinkable source of alternative and active agents against *T. gondii*.

## Introduction

*Toxoplasma gondii*, an obligate intracellular protozoan prevalent in one-third of the world’s population, has been thought of as relevant to public health mainly within the context of postnatally acquired infection in immunocompromised patients or congenital toxoplasmosis (Torgerson et al., 2015; Milne et al., 2020). Human infection could be acquired from food or water contaminated with tissue cysts and/or oocysts (Attias et al., 2020). Within the immunocompetent individual, while the host’s immunity has a positive effect on suppression of the proliferation of tachyzoite, it fails to robustly eliminate parasites (Carlier et al., 2012). Therefore, although chronic infections are mainly subclinical, they often reactivate from bradyzoite to tachyzoite in patients who are immunocompromised (such as HIV-infected patients and immunosuppressed organ transplant patients) resulting in high mortality. Importantly, due to the ability of *T. gondii* to infect almost all warm-blooded animals, infection in animals, especially livestock, not only causes the loss of livestock economy but also makes them an important infection source (Li et al., 2015; Hill and Dubey 2016).

Despite decades of research, however, a few advances have been made in the treatment of toxoplasmosis (Dunay et al., 2018). To date, the combination of sulfadiazine and pyrimethamine is still the standardized treatment regimen for *T. gondii* infection (Neville et al., 2015). Although both drugs are available to target the tachyzoite, the active stage of infection, their deficiency in eliminating *T. gondii* cysts, which contain a lot of bradyzoites, is responsible for induction of chronic infection. Also, long-term medication could lead to severe hematological side effects and induce drug resistance (Montazeri et al., 2018; Ben-Harari et al., 2017). Consequently, the limited options of available treatment for toxoplasmosis patients underscore the urgent need for searching for novel, more efficient, and safe therapeutics for *T. gondii* infection.

In recent years, more attention has been paid to discover safer and more effective drugs for toxoplasmosis treatment due to some deficiencies in current drugs with severe side effects, parasite resistance, and expensive drug prices. Natural products showing reduced side effects have attracted increasing attention because of their chemosensitizing activities and synergistic effects. Marine natural products refer to compounds isolated from marine microorganisms and phytoplankton, algae, sponges, cnidarians, bryozoans, molluscs, tunicates, echinoderms, mangroves, and other intertidal plants and microorganisms (Blunt et al., 2015). Several marine natural products, such as polysaccharides, steroids, fatty acids, carotenoids, halogenated compounds, and peptides, have exhibited a large number of biological properties including anticancer (Blunt et al., 2015; Saadaoui et al., 2020) and antiprotozoan activity (Álvarez-Bardón et al., 2020; Nweze et al., 2021; Yoo et al., 2020; Sanchez et al., 2013). Therefore, it is worthwhile to study the efficacy of marine products in the treatment of toxoplasmosis.

Zhejiang and Shandong provinces, with a superior ocean environment, are rich in marine resources. In this study, a library of 103 marine natural products was screened to identify those that suppress *T. gondii* growth *in vitro*. Products with good anti-*T. gondii* activity and low host cell toxicity were further evaluated for their effects on tachyzoite division and proliferation *in vitro*.

## Materials and Methods

### Chemicals

The Marine Natural Product Library containing 103 compounds was obtained from the Natural Product Library (Cat #L1400; Selleck, Shanghai, China) by searching the Dictionary of Marine Natural Products in the ChemNetBase website (https://dmnp.chemnetbase.com/faces/chemical/ChemicalSearch.xhtml) and applied for preliminary screens against *T. gondii*. Estradiol benzoate (Cat #S4110; Selleck) and octyl gallate (Cat #S9338; Selleck) were also obtained from Selleck. These compounds were dissolved with dimethyl sulfoxide (DMSO) into a 10 mM solution and stored at −20°C. Pyrimethamine (Cat # 46706, Sigma-Aldrich, Shanghai, China) was used as a reference drug. All information of compounds is listed in Supplementary Table S1.

### Parasites and Host Cells

*T. gondii* tachyzoites of the RH-2F strain, expressing luminescence-based β-galactosidase (β-Gal) (Seeber and Boothroyd, 1996), were maintained in monolayers of human foreskin fibroblasts (HFFs, ATCC SCRC-1041) cultured in Dulbecco’s modified Eagle’s medium (DMEM; Gibco, Invitrogen, Shanghai, China) made up of 10% (v/v) fetal bovine serum (FBS; Gibco, Invitrogen, Australia) and a cocktail of 1% (v/v) penicillin–streptomycin–glutamine (Cat # 10378016, Gibco) at 37°C and 5% CO_2_.

### Preliminary screen

To determine inhibition of parasite growth, β-Gal activity assay was performed in the preliminary screening as described previously (Liu et al., 2020). In brief, each compound was diluted fresh by using DMEM without phenol red to a final concentration of 10 μM and added to confluent HFFs in a 96-well half-area plate. Then mechanically released RH-2F tachyzoites were added at a multiplicity of infection (MOI) of 0.2 (parasite/host cell ratio). HFFs treated with 0.1% DMSO were used as the negative control, and 10 μM pyrimethamine was used as the positive control. After incubation for 72 h, 1 mM chlorophenol red-β-d-galactopyranoside (CPRG; Cat # 220588, Sigma-Aldrich) was added to the medium, and the absorbance was monitored at 560 nm. The number of parasites was calculated according to the standard curves made at the same time. Afterward, those compounds that displayed over 70% parasite inhibition ratio were further chosen to assess their cytotoxicity by using a CellTiter 96R AQueous One Solution Cell Proliferation assay system (Promega Corp, United States) according to manufacturer’s procedure. Compounds exhibiting low cytotoxicity (≥80% cell viability) were evaluated for inhibition efficacy in detail.

### *In vitro* Assessment of Drug Efficacy

Either estradiol benzoate or octyl gallate was added to the first column of HFFs (∼500 cells/well) in a 96-well half-area plate at a final concentration of 50 μM and then diluted serially across the plate by twofold, leaving the final column drug free. As a result, the exact concentration of each well was 50, 25, 12.5, 6.25, 3.125, 1.563, 0.781, 0.391, 0.195, and 0 μM, respectively. Fresh extracellular tachyzoites were added to each well at an MOI of 0.2 and incubated at 37°C and 5% CO_2_ for 72 h. The absorbance was detected at 560 nm 24 h after addition of CPRG. Meanwhile, HFF cells (1 × 10^5^ cells/well) treated with different concentrations of each compound were cultured in a 96-well plate at 37°C and 5% CO_2_ for 72 h, and then their cytotoxicity was assessed as described above. The exact concentration of the estradiol benzoate was 500, 250, 125, 62.5, 31.25, 15.625, 7.813, 3.906, 1.953, and 0 μM. The exact concentration of the octyl gallate was 100, 50, 25, 12.5, 6.25, 3.125, 1.563, 0.781, 0.391, and 0 μM. The assay was performed in triplicate and repeated three times. The 50% toxicity dose to host cell (TD_50_) and 50% inhibitory concentration to the parasite (IC_50_) were calculated, and the therapeutic index (TI) of each compound was defined as TD_50_/IC_50_.

### Plaque Assay

The plaque assay was performed as described previously (Liu et al., 2020). Monolayers of HFF in 24-well plates containing 5 μM of either estradiol benzoate or octyl gallate in triplicate were infected with freshly released 100 tachyzoites. DMSO-treated parasites were used as negative control, and the 10 mM pyrimethamine-treated group was used as positive control. Seven days after incubation, the wells were fixed with methanol and stained with 1.5% crystal violet. The plaque area of each group was measured with ImageJ software.

### Invasion Assay

The invasion assay was performed as described previously (Wilson et al., 2020) with minor modifications. RH-2F parasites were inoculated in HFFs and pretreated with 5 μM of each compound for 8 h. Then the freshly released tachyzoites (5 × 10^6^) were added to a new 24-well plate of HFFs with coverslips and each compound. Plates were kept on ice for 15 min and subsequently put at 38°C for 30 min to allow the parasites to invade host cells. Infected cells fixed with 4% paraformaldehyde were stained with mouse anti-*T. gondii* SAG1 (1:5,000, Cat # ab8313; Abcam, United States) for 1 h followed by antimouse Alexa Fluor 488 (1:1000, Invitrogen, Shanghai, China) for 1 h. Next, cells were permeabilized with 0.25% Triton-X100 and blocked with 3% BSA. An incubation step for 1 h with mouse anti-SAG1 was done followed by staining with anti-mouse Alexa Fluor 594 (1:1,000, Invitrogen, Shanghai, China) for 1 h. Parasite and host cell nuclei were then stained with DAPI (4′,6-diamidino-2-phenylindole) for 5 min. Intracellular parasites were shown as green^−^/red^+^, and the extracellular parasites were shown as green^+^/red^+^ (yellow). The proportion of the number of intracellular parasites to the total number of tachyzoites was termed as the invasion ratio. Ten fields were randomly selected under fluorescent microscope for counting, and three repetitions were performed.

### Intracellular Proliferation Assay

The intracellular proliferation assay was performed as described previously (Liu et al., 2020). Monolayers of HFF in 24-well plates were infected with fresh tachyzoites at MOI = 4. After 3 h, the medium was replaced with fresh culture medium containing 5 μM of each compound. Then 24 h later, the cells were fixed, permeabilized, and stained with mouse anti-SAG1 for 1 h followed by anti-mouse Alexa Fluor 594 for 1 h. At least 100 parasitophorous vacuoles were randomly selected, and the number of parasites in each vacuole was counted.

### Egress assay

The egress assay was performed as described previously (Wilson et al., 2020) with minor modifications. Monolayers of HFF in 24-well plates were infected with 1 × 10^4^ tachyzoites followed by incubation at 37°C and 5% CO_2_ in a medium containing 5 μM of each compound for 24 h. Prior to egress assays, infected wells were washed three times with prewarmed PBS. After the final wash, the cells were treated with 3 μM A23187. The treated plates were allowed to incubate at 5% CO_2_ and 37°C for 5 min before addition of 100% methanol and immediate placement on ice for 10 min. Subsequently, IFA assay was carried out as described in the invasion assay, and the proportion of the number of egressed parasites to the total number of tachyzoites was termed as the egress ratio. Ten fields were randomly selected under fluorescent microscope for counting, and two repetitions were performed.

### Flow Cytometric Analyses of the Cell Cycle

Flow cytometric analyses was performed as described previously (Liu et al., 2020). Monolayers of HFF were infected with tachyzoites at MOI = 1. After 12 h, synchronization of cell cycle was performed with 80 μM pyrrolidine dithiocarbamate (PDTC, Cat #S1809; Beyotime) at 37°C and 5% CO_2_ for 8 h. Then the medium was replaced with culture medium in the presence of 5 μM of each compound. After 8 h, the extracellular parasites were removed by cold PBS washing, and the intracellular tachyzoites were isolated from host cells through a 27-gauge needle and purified with a 3-μm filter. After centrifugation at 4°C for 10 min at 1,000 × g, parasites were fixed with 70% ethanol at −20°C for 24 h followed by cold 2% FBS washing. Parasites were stained with propidium iodide (PI; Cat # PH0530, Phygene) at 37°C for 30 min in the dark and then were filtered with a 5-μm pore filter. Harvested parasites were detected on FACSCanto™II flow cytometer (Beckman Coulter, CA, United States), and the results were analyzed using FlowJo 7.6.1 software (FlowJo LLC, Ashland, OR, United States). At least 50,000 events were collected per sample.

### Statistical Analysis

GraphPad Prism 8.0 software (GraphPad Software Inc., San Diego, CA, United States) was used to analyze all the data. Data were plotted and are presented as the mean of three replicates ± standard deviation (SD). The TD_50_ and IC_50_ values were plotted through a nonlinear regression analysis (curve fit). Phenotypic assay data were analyzed with two-tailed Student’s *t*-test or one-way ANOVA with Tukey’s *post-hoc* test. A value of *p* < 0.05 was considered statistically significant.

## Results

### Preliminary screening identified two anti-*Toxoplasma* compounds

**FIGURE 1:**
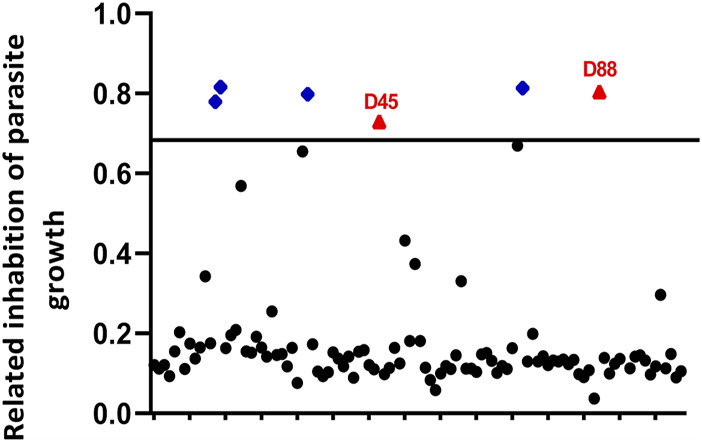
Primary small-molecule screening of different compounds that show anti-*Toxoplasma gondii* activity and less cytotoxicity. Human foreskin fibroblast (HFF) cells infected with RH-2F were cultured with 10 μM of each compound for 72 h, and then parasite growth was detected by β-Gal activity. The dimethyl sulfoxide (DMSO) group was used as negative control group to calculate the inhibition rate. In addition, among the compounds that inhibited parasite growth more than 70%, we screened the compounds with less cytotoxicity (≥80% cell viability). Red triangles, no host cell toxicity (D45 refers to estradiol benzoate; D88 refers to octyl gallate); blue diamonds, host cell toxicity; black circles, less than 70% parasite inhibition.

We screened 103 marine natural products to identify novel drugs that were anti-*T. gondii* (Supplementary Table S1). Our selection criteria aimed to identify compounds that caused parasite growth inhibition (at least 70% inhibition ratio) and had low cytotoxicity toward the host cell (at least 80% cell viability ratio). Two compounds, estradiol benzoate (parasite inhibition ratio: 73.0 ± 0.9%; host viability ratio: 136.4 ± 2.5%) and octyl gallate (parasite inhibition ratio: 80.4 ± 1.0%; host viability ratio: 98.6 ± 8.8%), matched our criteria (Figure 1).

### Evaluation of the Anti-*Toxoplasma* Activity and Cytotoxicity of Two Compounds

**FIGURE 2:**
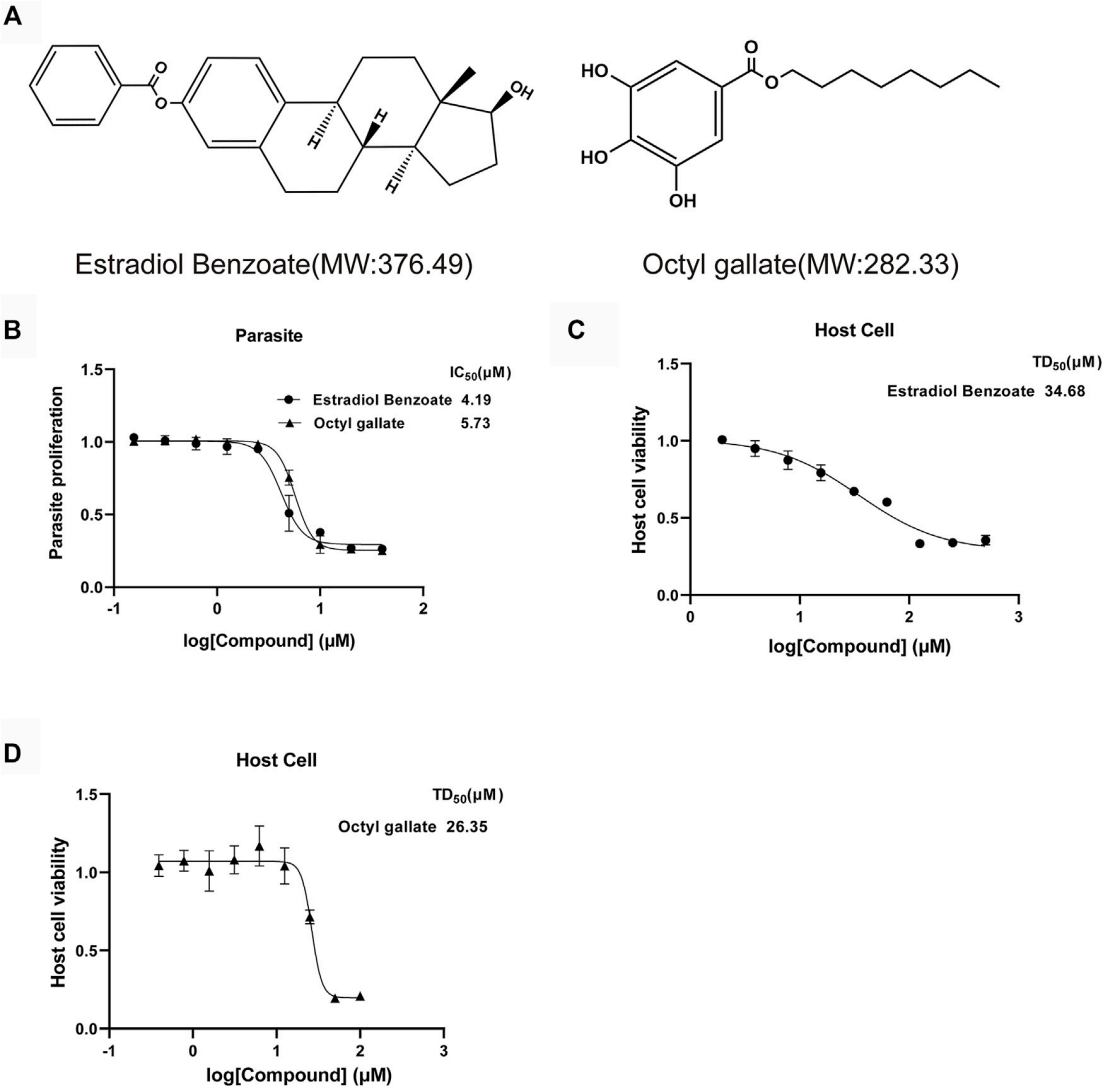
Estradiol benzoate and octyl gallate have dose-dependent inhibitory effect on *T. gondii* growth. **(A)** Molecular structures and molecular weights of estradiol benzoate and octyl gallate. **(B)** Dose–response response curves and IC_50_ values for estradiol benzoate and octyl gallate. RH-2F tachyzoite-infected HFFs were incubated with serial concentrations (0–50 μM) of the test compounds, and β-Gal activity was measured to evaluate parasite growth. **(C)** TD_50_ values of estradiol benzoate on HFF cells. HFF cells were incubated with various concentrations (0–500 μM) of estradiol benzoate for 72 h, and its cytotoxicity was evaluated. **(D)** TD_50_ values of octyl gallate on HFF cells. HFF cells were incubated with various concentrations (0–100 μM) of octyl gallate for 72 h, and its cytotoxicity was evaluated.

To further evaluate the efficiency of estradiol benzoate and octyl gallate on anti-*T. gondii* activity, the growth inhibition of the parasite by estradiol benzoate and octyl gallate (Figure 2A) was tested against the RH-2F strain expressing β-gal, allowing colorimetric quantitation of *T. gondii.* The results showed that estradiol benzoate and octyl gallate inhibit parasites at IC_50_ values of 4.41 ± 0.94 and 5.66 ± 0.35 μM (estradiol benzoate vs. octyl gallate: *p* = 0.095), respectively (Figure 2B). To analyze the cytotoxicity of each compound on HFF host cells *in vitro*, we examined cell proliferation using an MTT assay. The TD_50_ values of estradiol benzoate and octyl gallate were found to be 34.11 ± 2.86 and 26.4 ± 0.98 μM (estradiol benzoate vs. octyl gallate: *p* = 0.012), respectively (Figures 2C,D). According to the results above, the *in vitro* TI was calculated to be 7.7 for estradiol benzoate and 4.7 for octyl gallate.

**FIGURE 3:**
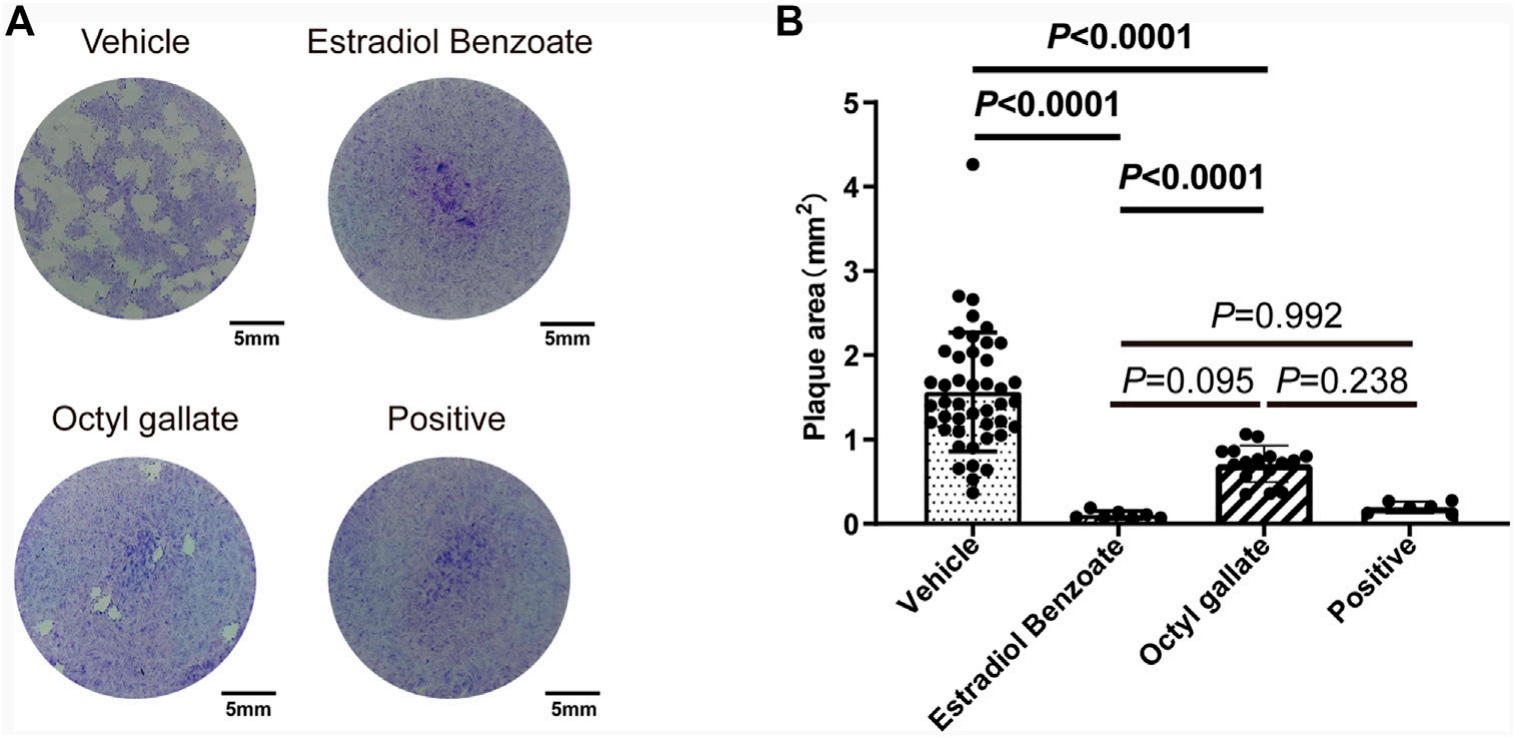
Effects of estradiol benzoate and octyl gallate on the lytic cycle of parasites. **(A)** HFF monolayers infected with 100 tachyzoites were treated with 5 μM of estradiol benzoate, octyl gallate, or pyrimethamine for 7 days. **(B)** Plaque areas were measured and shown as the mean ± SD from one experiment representative of three independent experiments.

Next, we performed a plaque assay to validate the effect of each compound on parasite and host cell. Seven days after incubation, we observed that the monolayer of HFF was intact, further indicating the low cytotoxicity of the two compounds. Importantly, the parasites treated with estradiol benzoate (0.11 ± 0.04) and octyl gallate (0.71 ± 0.21) displayed approximately 93% and 55% reductions in the plaque area compared with the vehicle control (1.56 ± 0.71), respectively (Figure 3). Also, we noticed that estradiol benzoate inhibition in forming of plaque is comparable with pyrimethamine (0.2 ± 0.07, 87% reduction in plaque area). The results suggest that the two compounds indeed inhibit the replication of tachyzoites.

### The Two Compounds Inhibit the Intracellular Growth of *Toxoplasma*

**FIGURE 4:**
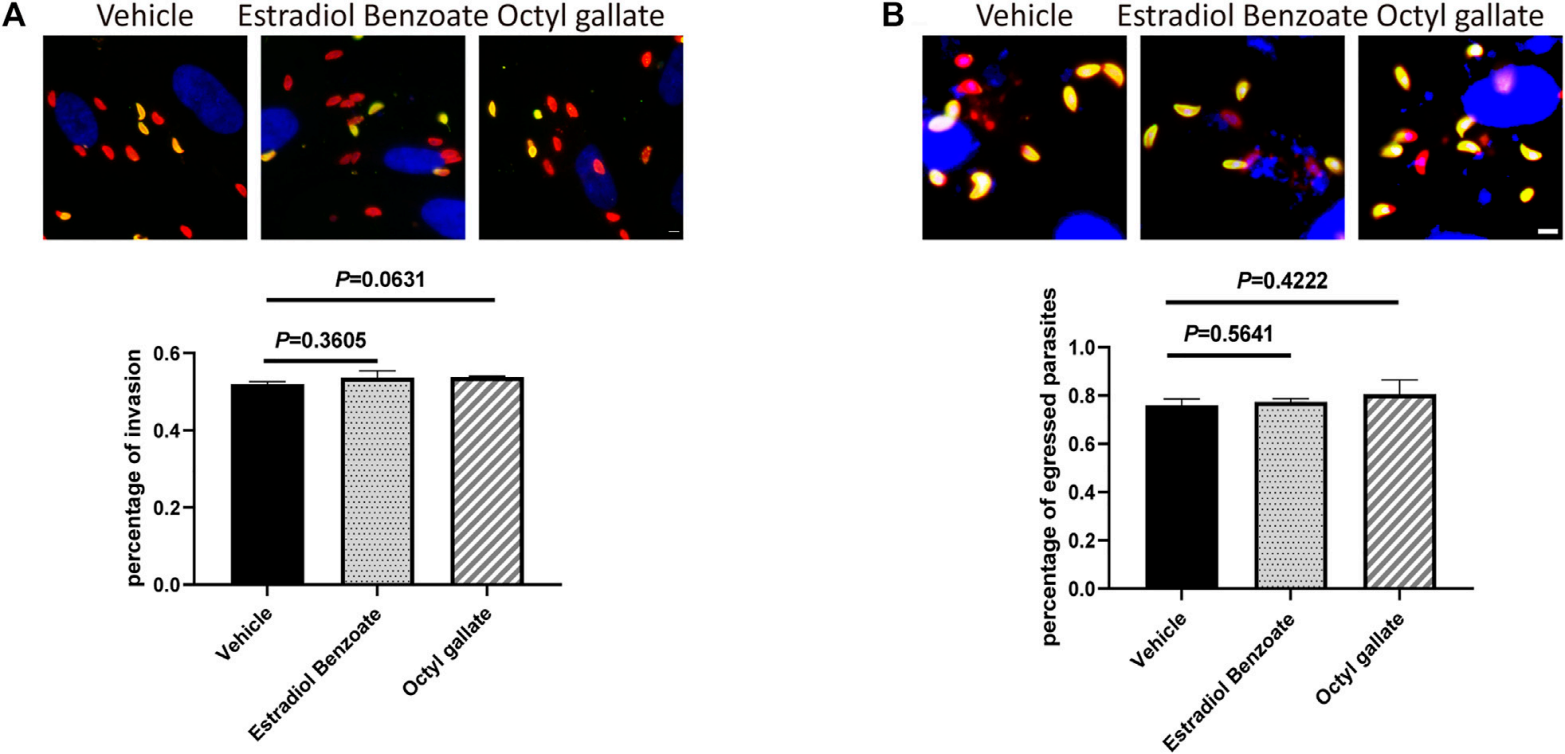
Estradiol benzoate and octyl gallate have no effect on invasion and egression of *T. gondii*. **(A)** Representative immunofluorescence micrographs of intracellular and extracellular parasites are shown, and the percentage of invaded parasites after treatment was calculated, respectively. **(B)** Representative immunofluorescence micrographs of egressed parasites are shown, and the percentage of egressed parasites was calculated. The mean ± SD from one experiment representative of three independent experiments was shown. Scale = 5 μm.

**FIGURE 5:**
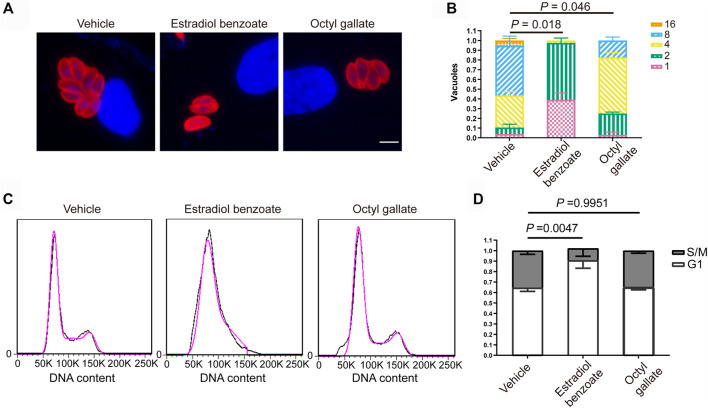
Inhibitory effects of estradiol benzoate and octyl gallate on parasite intracellular replication. **(A)** HFFs infected with RH-2F were treated with estradiol benzoate, octyl gallate, or DMSO for 24 h. The cells were fixed and detected using IFA. Immunofluorescence micrographs of intracellular parasites are shown. **(B)** The number of parasites per vacuole were counted. The percentage of vacuoles containing different numbers of parasites was shown as the mean ± SD from one experiment representative of three independent experiments. Scale = 5 μm. **(C)** Flow cytometric analysis of the effects of estradiol benzoate and octyl gallate on cell cycle progression. Propidium iodide (PI) staining was used to measure the DNA contents. **(D)** The percentage of parasites in the S/M and G1 phases of the cell cycle.

To unravel the mode of the anti-*T. gondii* action of the two compounds, we sought to determine whether any step of the lytic cycle of the parasite was impaired. We found that both estradiol benzoate and octyl gallate did not robustly suppress the ability of parasites to invade (estradiol benzoate: 0.52 ± 0.01; octyl gallate: 0.54 ± 0.02; vehicle control: 0.54 ± 0.01) and egress (estradiol benzoate: 0.77 ± 0.01; octyl gallate: 0.81 ± 0.06; vehicle control: 0.76 ± 0.03) (Figure 4). However, both compounds displayed potent ability to inhibit tachyzoite intracellular growth, as shown by the accumulation of vacuoles with mostly one, two, or four tachyzoites (Figures 5A,B). By contrast, the parasites treated with DMSO were mainly composed of vacuoles containing eight tachyzoites (DMSO: 0.51 ± 0.10; estradiol benzoate: 0 ± 0; octyl gallate: 0.18 ± 0.04).

### Estradiol Benzoate Inhibits the Cell Cycle of Tachyzoites

To clarify their potential mode of anti-*T. gondii* action, we sought to investigate the effect of the two compounds on parasite cell cycle progression by using flow cytometry. We noticed that estradiol benzoate (0.91 ± 0.08), rather than octyl gallate (0.65 ± 0.02), markedly resulted in a G1 accumulation peak compared with the mock treatment (0.64 ± 0.04) (Figures 5C, D), demonstrating that estradiol benzoate deferred or halted the cell cycle of tachyzoites by stumbling progression from G1 to S phase.

## Discussion

In this study, 103 marine natural products were screened and evaluated to discover novel marine-derived anti-*T. gondii* agents, and two compounds, estradiol benzoate and octyl gallate, were identified with high anti-*T. gondii* activity and low cytotoxicity. In addition, the key to the development and eventual utilization of anti-*T. gondii* compounds will be to maximize host cell selectivity. To this end, therapeutic index was used to assess the selectivity of both compounds. We found that the improved selectivity of estradiol benzoate and octyl gallate against *T. gondii* over host cells was paralleled by an approximate five- and eightfold greater potency, respectively. Importantly, the plaque assay revealed that the inhibitory activity of both compounds was comparable with that of pyrimethamine (Figure 3B). A previous investigation also showed that the TI value of sulfadiazine and pyrimethamine, which are currently used in the clinical treatment of toxoplasmosis, was ≤1 and ≤8, respectively (Kamau et al., 2012). Taken together, these findings suggest that the two compounds, especially estradiol benzoate, may be desirable agents for *T. gondii* therapy.

Estradiol benzoate is an estradiol analog that contains a benzyl ester at the C-3 position, and the compound binds to estrogen receptor α (ERα) in humans and mice (Matthews et al., 2000). The role of estradiol is not fully understood, but studies have shown that estradiol plays an important role in neurodegenerative diseases in an anti-inflammatory fashion (Marin and Diaz, 2018; Aguirre-Vidal et al., 2017; Sahab-Negah et al., 2020), since estradiol can upregulate the synthesis and secretion of inflammatory factors, e.g., TNFα, CXCL-8, IL-8, and IL-6 (Aguirre-Vidal et al., 2020). In addition, estradiol can regulate the estrogen receptor and growth factor signaling pathways to induce apoptosis (Hossain et al., 2021). In our phenotypic experiments, estradiol benzoate was also found to arrest the cell cycle and thereby inhibit tachyzoite proliferation. However, a previous *in vivo* study showed that the addition of estradiol benzoate to either atovaquone-treated group or the combination treatment of metronidazole and spiramycin did not exhibit more effect in reducing the brain cyst burden in *T. gondii*-infected male mice (Hegazy et al., 2019). Nevertheless, due to the excellent inhibition efficiency in parasite growth and low cytotoxicity to host cell, we think that the estradiol benzoate treatment conditions, such as dose, period, and route of administration, needed to be further optimized.

Octyl gallate (3,4,5-trihydroxybenzoate), an alkyl gallate, is a widely used food additive and has been reported to possess broad antimicrobial (Fujita and Kubo, 2002; Saibabu et al., 2020; Uozaki et al., 2007) and anticancer (Sales et al., 2018) activities. However, there is a different mechanism of octyl gallate in antifungal and antiviral activities. The antifungal activity mainly associates with its antioxidant activity, which causes fungal mitochondrial dysfunction and subsequently increase intracellular reactive oxygen species (ROS) level (Saibabu et al., 2020), while virucidal activity of octyl gallate may contribute to directly inactivate a virus by inhibition of the transcriptional and/or genomic replication of the virus (Uozaki et al., 2007; Yamasaki et al., 2007). In this study, we found that octyl gallate effectively inhibits parasite intracellular growth. By the combination of intact morphology of host cell observed through plaque assay and high TI value, we speculate that octyl gallate suppresses parasite intracellular growth by hindering a parasite rather than a host target, though further research is needed to confirm that.

Despite both estradiol benzoate and octyl gallate exhibiting outstanding antiparasitic activity, the corresponding target of both compounds in *T. gondii* has not been identified. Consequently, the exact mechanism is still unclear and needs to be investigated next. On this basis, it is necessary to study their toxicology and optimize their structure. Meanwhile, due to the lack of type II strain of *Toxoplasma*, an avirulent strain that can be induced to form cysts *in vitro*, we cannot evaluate whether both compounds are available to inhibit the formation of cyst or eliminate bradyzoite. Finally, to assess the therapeutic effect of the two compounds in mice infected with *T. gondii*, it is necessary to determine the concentration and duration of treatment.

In brief, we identified two targets with anti-*T. gondii* activity and low cytotoxicity from a library of 103 natural marine products. Although the mechanism of action against *T. gondii* clearly needs further investigation, available data suggest that these two products are hopeful candidates for the treatment of toxoplasmosis.

## Data Availability

The original contributions presented in the study are included in the article/Supplementary Material, further inquiries can be directed to the corresponding authors.

## References

[B1] Aguirre-Vidal Y. Monroy-Noyola A. Anaya-Ramos L. Arteaga-Silva M. Mendez-Armenta M. Ostoa-Saloma P. (2017). β-Estradiol-3-benzoate Confers Neuroprotection in Parkinson MPP+ Rat Model through Inhibition of Lipid Peroxidation. Steroids 126, 7–14. 10.1016/j.steroids.2017.08.001 28827046

[B2] Aguirre-Vidal Y. Morales-Montor J. Gómez de León C. T. Ostoa-Saloma P. Díaz-Zaragoza M. Montes S. (2020). Protection Induced by Estradiol Benzoate in the MPP+ Rat Model of Parkinson's Disease Is Associated with the Regulation of the Inflammatory Cytokine Profile in the Nigro Striatum. J. Neuroimmunol 349, 577426. 10.1016/j.jneuroim.2020.577426 33096292

[B3] Álvarez-Bardón M. Pérez-Pertejo Y. Ordóñez C. Sepúlveda-Crespo D. Carballeira N. M. Tekwani B. L. (2020). Screening marine Natural Products for New Drug Leads against Trypanosomatids and Malaria. Mar. Drugs 18, 187. 10.3390/md18040187 PMC7230869 32244488

[B4] Attias M. Teixeira D. E. Benchimol M. Vommaro R. C. Crepaldi P. H. De Souza W. (2020). The Life-Cycle of *Toxoplasma Gondii* Reviewed Using Animations. Parasit Vectors 13, 588. 10.1186/s13071-020-04445-z 33228743 PMC7686686

[B5] Ben-Harari R. R. Goodwin E. Casoy J. (2017). Adverse Event Profile of Pyrimethamine-Based Therapy in Toxoplasmosis: a Systematic Review. Drugs R. D 17, 523–544. 10.1007/s40268-017-0206-8 28879584 PMC5694419

[B6] Blunt J. W. Copp B. R. Keyzers R. A. Munro M. H. Prinsep M. R. (2015). Marine Natural Products. Nat. Prod. Rep. 32, 116–211. 10.1039/c4np00144c 25620233

[B7] Carlier Y. Truyens C. Deloron P. Peyron F. (2012). Congenital Parasitic Infections: a Review. Acta Trop. 121, 55–70. 10.1016/j.actatropica.2011.10.018 22085916

[B8] Dunay I. R. Gajurel K. Dhakal R. Liesenfeld O. Montoya J. G. (2018). Treatment of Toxoplasmosis: Historical Perspective, Animal Models, and Current Clinical Practice. Clin. Microbiol. Rev. 31, e00057–17. 10.1128/CMR.00057-17 30209035 PMC6148195

[B9] Fujita K. Kubo I. (2002). Antifungal Activity of Octyl Gallate. Int. J. Food Microbiol. 79, 193–201. 10.1016/s0168-1605(02)00108-3 12371654

[B10] Hegazy M. M. Elmehankar M. S. Azab M. S. El-Tantawy N. L. Abdel-Aziz A. (2019). Sex Dichotomy in the Course of Experimental Latent Toxoplasmosis. Exp. Parasitol. 202, 15–21. 10.1016/j.exppara.2019.05.003 31078550

[B11] Hill D. E. Dubey J. P. (2016). *Toxoplasma Gondii* as a Parasite in Food: Analysis and Control. Microbiol. Spectr. 4. 10.1128/microbiolspec.PFS-0011-2015 27726776

[B12] Hossain M. S. Quadery Tonmoy M. I. Islam M. N. Islam M. S. Afif I. K. Singha Roy A. (2021). MicroRNAs Expression Analysis Shows Key Affirmation of Synaptopodin-2 as a Novel Prognostic and Therapeutic Biomarker for Colorectal and Cervical Cancers. Heliyon 7, e07347. 10.1016/j.heliyon.2021.e07347 34195444 PMC8239731

[B13] Kamau E. T. Srinivasan A. R. Brown M. J. Fair M. G. Caraher E. J. Boyle J. P. (2012). A Focused Small-Molecule Screen Identifies 14 Compounds with Distinct Effects on *Toxoplasma Gondii* . Antimicrob. Agents Chemother. 56, 5581–5590. 10.1128/AAC.00868-12 22908155 PMC3486605

[B14] Li Y. N. Nie X. Peng Q. Y. Mu X. Q. Zhang M. Tian M. Y. (2015). Seroprevalence and Genotype of *Toxoplasma Gondii* in Pigs, Dogs and Cats from Guizhou Province, Southwest China. Parasit Vectors 8, 214. 10.1186/s13071-015-0809-2 25889417 PMC4394553

[B15] Liu S. Wu M. Hua Q. Lu D. Tian Y. Yu H. (2020). Two Old Drugs, NVP-Aew541 and GSK-J4, Repurposed against the *Toxoplasma Gondii* RH Strain. Parasit Vectors 13, 242. 10.1186/s13071-020-04094-2 32393321 PMC7216583

[B16] Marin R. Diaz M. (2018). Estrogen Interactions with Lipid Rafts Related to Neuroprotection. Impact of Brain Ageing and Menopause. Front. Neurosci. 12, 128. 10.3389/fnins.2018.00128 29559883 PMC5845729

[B17] Matthews J. Celius T. Halgren R. Zacharewski T. (2000). Differential Estrogen Receptor Binding of Estrogenic Substances: a Species Comparison. J. Steroid Biochem. Mol. Biol. 74, 223–234. 10.1016/s0960-0760(00)00126-6 11162928

[B18] Milne G. Webster J. P. Walker M. (2020). Toxoplasma Gondii: AnUnderestimated Threat? Trends Parasitol. 36, 959–969. 10.1016/j.pt.2020.08.005 33012669

[B19] Montazeri M. Mehrzadi S. Sharif M. Sarvi S. Tanzifi A. Aghayan S. A. (2018). Drug Resistance in *Toxoplasma Gondii* . Front. Microbiol. 9, 2587. 10.3389/fmicb.2018.02587 30420849 PMC6215853

[B20] Neville A. J. Zach S. J. Wang X. Larson J. J. Judge A. K. Davis L. A. (2015). Clinically Available Medicines Demonstrating Anti-*toxoplasma* Activity. Antimicrob. Agents Chemother. 59, 7161–7169. 10.1128/AAC.02009-15 26392504 PMC4649158

[B21] Nweze J. A. Mbaoji F. N. Li Y. M. Yang L. Y. Huang S. S. Chigor V. N. (2021). Potentials of marine Natural Products against Malaria, Leishmaniasis, and Trypanosomiasis Parasites: a Review of Recent Articles. Infect. Dis. Poverty 10, 9. 10.1186/s40249-021-00796-6 33482912 PMC7821695

[B22] Saadaoui I. Rasheed R. Abdulrahman N. Bounnit T. Cherif M. Al Jabri H. (2020). Algae-derived Bioactive Compounds with Anti-lung Cancer Potential. Mar. Drugs 18, 197. 10.3390/md18040197 PMC7230368 32276401

[B23] Sahab-Negah S. Hajali V. Moradi H. R. Gorji A. (2020). The Impact of Estradiol on Neurogenesis and Cognitive Functions in Alzheimer's Disease. Cell Mol Neurobiol 40, 283–299. 10.1007/s10571-019-00733-0 31502112 PMC11448899

[B24] Saibabu V. Fatima Z. Ahmad K. Khan L. A. Hameed S. (2020). Octyl Gallate Triggers Dysfunctional Mitochondria Leading to ROS Driven Membrane Damage and Metabolic Inflexibility along with Attenuated Virulence in *Candida Albicans* . Med. Mycol. 58, 380–392. 10.1093/mmy/myz054 31135913

[B25] Sales M. S. Roy A. Antony L. Banu S. K. Jeyaraman S. Manikkam R. (2018). Octyl Gallate and Gallic Acid Isolated from *Terminalia Bellarica* Regulates normal Cell Cycle in Human Breast Cancer Cell Lines. Biomed. Pharmacother. 103, 1577–1584. 10.1016/j.biopha.2018.04.182 29864945

[B26] Sanchez L. M. Knudsen G. M. Helbig C. De Muylder G. Mascuch S. M. Mackey Z. B. (2013). Examination of the Mode of Action of the Almiramide Family of Natural Products against the Kinetoplastid Parasite *Trypanosoma Brucei* . J. Nat. Prod. 76, 630–641. 10.1021/np300834q 23445522 PMC3971013

[B27] Seeber F. Boothroyd J. C. (1996). *Escherichia coli* Beta-Galactosidase as an *In Vitro* and *In Vivo* Reporter Enzyme and Stable Transfection Marker in the Intracellular Protozoan Parasite *Toxoplasma Gondii* . Gene 169, 39–45. 10.1016/0378-1119(95)00786-5 8635747

[B28] Torgerson P. R. Devleesschauwer B. Praet N. Speybroeck N. Willingham A. L. Kasuga F. (2015). World Health Organization Estimates of the Global and Regional Disease burden of 11 Foodborne Parasitic Diseases, 2010: A Data Synthesis. Plos Med. 12, e1001920. 10.1371/journal.pmed.1001920 26633705 PMC4668834

[B29] Uozaki M. Yamasaki H. Katsuyama Y. Higuchi M. Higuti T. Koyama A. H. (2007). Antiviral Effect of Octyl Gallate against DNA and RNA Viruses. Antivir. Res 73, 85–91. 10.1016/j.antiviral.2006.07.010 16950523

[B30] Wilson S. K. Heckendorn J. MartorelliDi Genova B. Koch L. L. Rooney P. J. Morrissette N. (2020). A *Toxoplasma Gondii* Patatin-like Phospholipase Contributes to Host Cell Invasion. Plos Pathog. 16, e1008650. 10.1371/journal.ppat.1008650 32628723 PMC7365478

[B31] Yamasaki H. Uozaki M. Katsuyama Y. Utsunomiya H. Arakawa T. Higuchi M. (2007). Antiviral Effect of Octyl Gallate against Influenza and Other RNA Viruses. Int. J. Mol. Med. 19, 685–688. 10.3892/ijmm.19.4.685 17334645

[B32] Yoo E. Schulze C. J. Stokes B. H. Onguka O. Yeo T. Mok S. (2020). The Antimalarial Natural Product Salinipostin A Identifies Essential α/β Serine Hydrolases Involved in Lipid Metabolism in P. falciparum Parasites. Cell Chem Biol 27, 143–e5. 10.1016/j.chembiol.2020.01.001 31978322 PMC8027986

